# Credibility of AI generated and human video doctors and the relationship to social media use

**DOI:** 10.3389/fpubh.2025.1559378

**Published:** 2025-07-09

**Authors:** Tao Liu, Peijia Wang, Deyin Pan, Ruixin Liu

**Affiliations:** ^1^Department of Cardiovascular Medicine, The Third Xiangya Hospital, Central South University, Changsha, China; ^2^Faculty of Clinical Medicine, Changsha Medical University, Changsha, Hunan, China; ^3^School of Journalism and Communication, Hunan University, Changsha, China; ^4^School of Journalism and Information Communication, Huazhong University of Science and Technology, Wuhan, China; ^5^Guoyitang Clinic, Beijing University of Chinese Medicine, Beijing, China

**Keywords:** social media use, artificial intelligence doctors, human doctors, online health video, age stereotype

## Abstract

**Objective:**

It’s unclear if the age stereotype of young doctors also applies to artificial intelligence (AI) doctors. Although research shows social media can reduce discrimination, age stereotypes are still underexplored. This study is aimed to determine the relationship between social media use and age stereotypes among doctors in online health videos narrated by AI/human doctors.

**Methods:**

This is a cross-sectional study, divided into two phases and conducted from May 25 to June 19, 2024. Self-reported questionnaire was developed and collected by face-to-face interview. All individuals who are 18 years old or above with adequate reading comprehension skills are eligible. The credibility of doctors among participants in online health videos in AI and human conditions and their relationship with the intensity of social media use were investigated. Univariable and multivariable generalized linear models were used to explore the relationship between social media use and age stereotypes.

**Results:**

We obtained 294 and 300 valid questionnaires in phase I and phase II, respectively. In both AI and human conditions, there is a preference for health education conducted by older doctors. Older doctors were rated the most credible (median score 14·00, IQR [12·00, 15·00] in the condition of AI, median score 14·00, IQR [12·00, 15·00] in the condition of Human). Both univariable and multivariable generalized linear models revealed a significant negative association between social media use and age stereotypes, particularly between older and younger doctors (*β* = −0·34, *p* < 0.001). In the condition of AI. In the condition of human, the intensity of social media use is not related to participants’ age stereotypes.

**Conclusion:**

The image of AI doctors can help patients avoid being influenced by age stereotypes, enabling them to evaluate doctors’ medical expertise more objectively.

## 1 Introduction

The integration of artificial intelligence (AI) into healthcare has led to the emergence of AI doctors, particularly in the realm of telemedicine and online health communication. These AI doctors, trained on vast medical datasets and enhanced through continual machine learning, are now featured in health videos across various social media platforms, offering basic medical knowledge, treatment suggestions, and personalized advice. Especially with the development of video-based generative AI, AI doctors narrated health content has become a popular medium for public health education, providing engaging visual experiences that enhance attention and comprehension (1–3).

However, while the technological benefits of AI doctors have been widely acknowledged from the perspective of patient services (4), little attention has been paid to their symbolic and communicative roles, particularly in how they reshape social perceptions of medical professionals. Studies suggest that stereotypes are often projected onto AI images, especially when they are designed to mimic human roles and identities (5, 6). Among these, age stereotypes are culturally-shared and deeply rooted in doctor-patient communication (7). Young doctors are often perceived as inexperienced compared to their older counterparts (7, 8), and older doctors are typically viewed as more credible in traditional health communication contexts (9). These age stereotypes not only undermine trust in young doctors but can also negatively affect their professional development, increasing stress and potentially damaging the doctor-patient relationship (10). Therefore, examining whether age stereotypes are present in AI doctors is theoretically significant and practically relevant for understanding how social biases may be perpetuated in seemingly neutral, technology-mediated health communication (11). As AI doctors increasingly participate in public health communication, especially on social media, they may influence not only patients’ knowledge acquisition but also their implicit attitudes toward medical professionals (12). Thus, uncovering the presence and dynamics of age stereotypes in AI-mediated health content is crucial for designing more equitable and inclusive health communication strategies.

Given that these videos are predominantly shared on social media platforms, meaning that users’ social media use will shape their perception (13). Social media use often plays a crucial role in altering stereotypes (14). However, whether it reduces or exacerbates stereotypes remains under investigation. One perspective is that it facilitates information exchange (15), challenges traditional biases, and promotes a more pro-social mindset (16). In other words, individuals who frequently use social media are less likely to hold stereotypes and express prejudice (17). Thus, we consider the intensity of social media use a potential factor in mitigating age stereotypes within online health videos, both in AI and human doctors.

Building on the above discussion, this study aims to investigate whether AI doctors are subject to age stereotypes in the context of online health videos. Specifically, we examined whether the intensity of social media use mitigating age stereotypes in both AI and human doctor conditions. To guide this inquiry, we propose the following research questions:

*RQ1*: Do age stereotypes persist in online health videos narrated by AI doctors?

*RQ2*: Does social media use mitigate the age stereotypes in both AI and human doctor narrated videos?

## 2 Methods

All methods were carried out in accordance with the STROBE statement for observational studies.

### 2.1 Ethics approval and consent to participate

This study was approved by the Ethics Review Committee of the Third Xiangya Hospital of Central South University (No. 24139) and was performed in accordance with the Declaration of Helsinki. Participants received an informed consent reminder before the start of the questionnaire.

### 2.2 Study location

The cross-sectional study was conducted in two phases in Changsha, China from May 25 to June 19, 2024, with participants receiving RMB 3 as a reward. The first phase examined the relationship between social media use and age stereotypes in health videos narrated by AI doctors, while the second phase focused on videos narrated by human doctors. Changsha stands out in medical digitalization, hosting China’s first Ministry Key Laboratory in Health Information Technology, specializing in mobile health, big data, AI, and the internet of things (18). Additionally, multiple hospitals in Changsha have integrated AI doctors as medical assistants (19). Therefore, we selected Changsha as the research site and conducted AI doctor survey using stratified sampling to ensure both real-world relevance and representativeness of this study. While our research was conducted in Changsha, it appears that the data collected may have the potential for broader cultural interpretation and could be extended to other settings (8, 20).

### 2.3 Study population

To ensure reliability and validity, with a 10% margin for invalid responses, the recommended sample size is at least 10 times the number of valid questions (21). With 23 valid questions in our questionnaire, a sample size of at least 256 participants per phase was estimated. To ensure adequacy, we aimed for a final sample size of 350 per phase.

A multistage stratified sampling approach was utilized. Initially, two districts, Yuelu and Tianxin, were chosen via a lottery system. District names were written on separate papers, placed in a container, and randomly drawn to ensure equal selection chances. Then, 30% of community health service centers in each district were randomly selected. This led to six centers in Yuelu (210 participants) and four in Tianxin (140 participants). In each selected center, participants were assigned unique identification numbers stored electronically. A computerized random selection was then conducted using these numbers to ensure equal selection probability for all eligible participants.

Eligible participants were adults aged 18 or older with adequate reading comprehension and social media experience, excluding those with severe clinical conditions like heart failure or acute coronary syndrome.

### 2.4 Process of questionnaire development

To establish the initial item pool, we searched five English databases (PubMed, Embase, Cochrane Library, EBSCO, and Web of Science) and four Chinese databases (CBM, CNKI, Wanfang Data, and VIP Information) using the keywords “social media use” “age stereotypes” and “artificial intelligence” After reviewing the literature, we compiled a pool of 35 questions, which was refined through expert consultation. The expert panel consisted of five specialists (three chief physicians and two directors, three males and two females, aged 35–55), each with over 10 years of experience and a doctoral degree in health communication or medical science. Two rounds of consultation were conducted. In the first round, items were modified, merged, or deleted based on expert feedback, while the second round focused on refining the items. A preliminary survey with 30 adults tested the questionnaire’s clarity and suitability. Ultimately, 12 of the 35 questions were excluded, resulting in a final questionnaire with 23 valid questions (Supplementary Questionnaires 1, 2).

### 2.5 Image generation

Preference for a specific narrator was assessed using a multiple-choice question with images. The two questionnaire phases differed only in this question: in phase I, AI-generated images of individuals aged 25, 40, and 60 were shown (created with “Midjourney V6” for younger and “Adobe Photoshop CC 2020” for older doctors). In phase II, real people with similar appearances but different ages (25, 40, and 60) were used. The younger doctor’s image was one of the authors, with permission granted for study use. To avoid bias, all images had consistent facial features, skin tones, and genders, with age labels for clarity. A manipulation check ensured participants could distinguish AI from human doctors. The videos covered common health topics, such as “How to dress during fever,” “Improving sleep quality,” “Myopia prevention,” “Measuring temperature and blood pressure,” and “Exercise tips.”

### 2.6 Measurement

#### 2.6.1 Intensity of social media use

We investigated the social media platforms most frequently used by participants (Including Weibo, WeChat, Douyin, Kuaishou, Bilibili, Xiaohongshu, Zhihu, QQ, Facebook, Instagram, TikTok, LinkedIn, YouTube, Snapchat, X/Twitter) and assessed the intensity of their use on these platforms. Social media use intensity was measured using eight self-reported questions, a scale widely recognized for its high construct validity in health communication research (22).

Participants’ social media use intensity was calculated by summing the scores for each question. For instance, if a participant has 180 friends (worth 4 points), uses the platform for 1·5 h per day (worth 3 points), and answers “strongly approve” (worth 5 points) to the remaining six questions, their total social media use intensity score would be 37. Details of the scale items are provided in Supplementary Table S1.

#### 2.6.2 Age stereotypes

Age stereotypes were assessed by understanding how a doctor’s age affects their perceived credibility in online health videos. If a doctor’s age positively correlates with their perceived credibility, it confirms age stereotypes. These stereotypes are relative (23), meaning a larger credibility gap between older and younger doctors indicates stronger age-related biases.

The perceived credibility scale used in this study is well-established in health communication research and has high construct validity (24). It includes three items, each scored from 5 (strongly approve) to 1 (strongly disapprove). The total score from these items reflects the participant’s perceived credibility of the doctor, as detailed in Supplementary Table S2.

#### 2.6.3 Other variables

When collecting data on participants’ medical conditions and family medical history, we focused on four major chronic diseases—hypertension, diabetes, dyslipidemia, and obesity—due to their significant health implications and high prevalence in the study population (25). Other illnesses were categorized as “others” Age was classified by quartiles, a method also used in various studies (26).

### 2.7 Data collection and analysis

#### 2.7.1 Data collection

Data were collected using a structured questionnaire administered through face-to-face interviews by a team of trained healthcare personnel. The interviews were conducted in a private setting to ensure confidentiality and comfort for the participants. Each participant was given ample time to complete the questionnaire, and the interviewers were available to clarify any doubts or questions that arose during the process.

The data were entered twice to detect and correct any discrepancies or missing entries using Epidata (version 3.1). After the initial data entry, a thorough data cleaning process was conducted to identify and handle outliers, inconsistencies, and missing data. Any missing data were excluded from the analysis to maintain the integrity of the dataset.

#### 2.7.2 Data analysis

Statistical analyses were conducted using R version 4.3.2. Categorical variables were presented as frequencies and percentages. The Kolmogorov–Smirnov test was employed to assess the normality of continuous data. Normally distributed continuous variables were reported as means ± standard deviations, while non-normally distributed variables were presented as medians with interquartile ranges (IQR). Given that the difference in perceived credibility was a continuous variable with a non-normal distribution, the relationship between social media use and age stereotypes was examined using both univariable and multivariable generalized linear models, with social media use serving as the independent variable and age stereotypes (difference in perceived credibility among doctors of different ages) as the dependent variable.

Age, health status, attitude toward online health videos, and variables significantly associated with age stereotypes in univariate generalized linear models (Supplementary Tables S3–S8) were selected as confounding variables for the multivariable generalized linear models, based on relevant literature and practical experience.

For sensitivity analysis, social media use intensity was dichotomized based on the median. Participants with usage above the median were classified as having high intensity, while those below the median were classified as having low intensity. Missing data were excluded from the analysis. A *p*-value of <0.05 was considered statistically significant, and all tests were two-tailed. Reliability of the questionnaire scales was evaluated using Cronbach’s *α* coefficient, while validity was assessed through Bartlett’s test of sphericity and the Kaiser–Meyer–Olkin (KMO) measure, confirming the robustness of the survey tool.

## 3 Results

### 3.1 Reliability and validity

In summary, the scales for assessing perceived credibility and the intensity of social media use, both in AI and human conditions, exhibit a certain degree of reliability and validity (Supplementary Tables S9, S10).

### 3.2 Status of age stereotypes

In phase I, 320 participants completed the questionnaires (response rate 91.4%). After excluding 26 incomplete responses, 294 (91.9%) valid questionnaires remained for analysis. Most participants (68.4%) were female, with the largest age group (25–35) comprising 26.5%. A total of 56.5% were married or living with a partner, and 36.1% had a monthly income of 3,000–8,000 yuan. Health status was rated average or good by 49.0% and 26.5%, respectively. Despite a 70.4% prevalence of family history of disease, actual rates of chronic conditions such as hypertension (11.2%), diabetes (3.4%), obesity (15.3%), and dyslipidemia (4.8%) were low. A positive attitude toward videos was reported by 44.2% of participants. The median social media use intensity was 25.00. Older doctors were rated the most credible (median score 14.00, IQR [12.00, 15.00]), followed by middle-aged doctors (12.00, IQR [11.0.00, 13.00]) and younger doctors (10.00, IQR [9.00, 12.00]) (Table 1).

**Table 1:** Characteristics of participants in the condition of AI doctors.

Variables	Level	Overall
Gender (%)	Male	93 (31.6)
	Female	201 (68.4)
Age (%)	16–24	78 (26.5)
	25–35	78 (26.5)
	35–43	67 (22.8)
	44–69	71 (24.1)
BMI (median [IQR])		21.36 [19.83, 23.87]
Education	Elementary school	25 (8.5)
	Junior high school	53 (18.0)
	Senior high school	56 (19.0)
	Associate degree	46 (15.6)
	Bachelor’s degree	75 (25.5)
	Master’s degree or above	39 (13.3)
Marital status (%)	Widowed/Divorced/Separated/Never married	128 (43.5)
	Married/Living with partner	166 (56.5)
Monthly income (%)	<3,000 yuan	71 (24.1)
	3,000–8,000 yuan	106 (36.1)
	8,000–20,000 yuan	97 (33.0)
	>20,000 yuan	20 (6.8)
Employment status (%)	Employment	214 (72.8)
	Unemployment	80 (27.2)
Health status (%)	Poor	72 (24.5)
	Average	144 (49.0)
	Good	78 (26.5)
Present disease (%)	No	212 (72.1)
	Yes	82 (27·9)
Family medical history (%)	No	87 (29·6)
	Yes	207 (70.4)
Attitude toward online health video (%)	Poor	46 (15.6)
	Average	118 (40.1)
	Good	130 (44.2)
Hypertension (%)	No	261 (88.8)
	Yes	33 (11.2)
Diabetes (%)	No	284 (96.6)
	Yes	10 (3.4)
Dyslipidemia (%)	No	280 (95.2)
	Yes	14 (4.8)
Obesity (%)	No	249 (84.7)
	Yes	45 (15.3)
Intensity of social media use (median [IQR])		25.00 [20.00, 29.00]
Perceived credibility of older doctors		14.00 [12.00, 15.00]
Perceived credibility of middle-aged doctors		12.00 [11.00, 13.00]
Perceived credibility of younger doctors		10.00 [9.00, 12.00]

**Figure 1:**
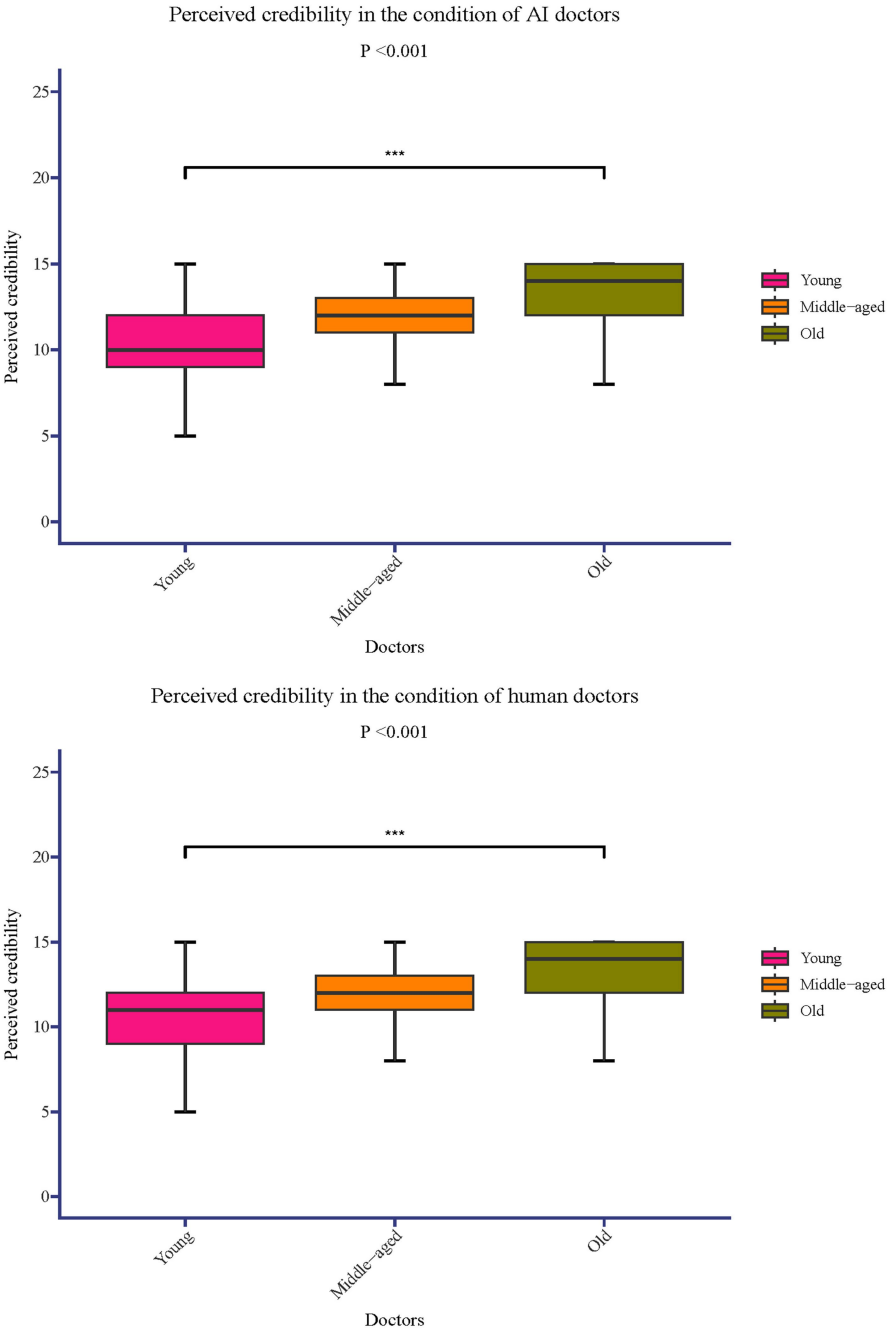
Perceived credibility in the condition of AI/human doctors.

The median difference in perceived credibility between older and younger doctors was 3.00 with an IQR of [1.00, 5.00] (*p* < 0.001). The difference between middle-aged and younger doctors had a median of 2.00 with an IQR of [0.00, 3.00] (*p* < 0.001) and the difference between older and middle-aged doctors was the smallest, with a median of 1.00 and an IQR of [0.00, 2.00] (*p* < 0.001) (Figure 1).

In phase II, 330 participants completed the questionnaires (response rate 94.3%). After excluding 30 incomplete responses, 300 (90.9%) valid questionnaires were analyzed. Of the participants, 68.3% were female, and the largest age group (25–33) comprised 26.7%. A total of 59.0% were married or living with a partner, and 37.0% earned 3,000–8,000 yuan monthly. Health status was rated as average by 57.3% and good by 23.3%. While 72.3% had a family history of disease, only 24.3% had chronic conditions, including hypertension (12.7%), diabetes (2.7%), obesity (11.0%), and dyslipidemia (5.3%). A positive attitude toward videos was reported by 55.0% of participants. The median social media use intensity was 32.00. Credibility ratings for the younger doctors had a median score of 11.00 (IQR [9.00, 12.00]), middle-aged doctors 12.00 (IQR [11.00, 13.00]), and the older doctors 14.00 (IQR [12.00, 15.00]) (Table 2).

**Table 2:** Characteristics of participants in in the condition of human doctors.

Variables	Level	Overall
Gender (%)	Male	95 (31.7)
	Female	205 (68.3)
Age (%)	18–24	77 (25.7)
	25–33	80 (26.7)
	34–43	74 (24.7)
	44–65	69 (23.0)
BMI (median [IQR])		21.35 [19.18, 23.68]
Education (%)	Elementary school	10 (3.3)
	Junior high school	70 (23.3)
	Senior high school	83 (27.7)
	Associate degree	47 (15.7)
	Bachelor’s degree	68 (22.7)
	Master’s degree or above	22 (7.3)
Marital status (%)	Widowed/Divorced/Separated/Never married	123 (41.0)
	Married/Living with partner	177 (59.0)
Monthly income (%)	<3,000 yuan	64 (21.3)
	3,000–8,000 yuan	111 (37.0)
	8,000–20,000 yuan	108 (36.0)
	>20,000 yuan	17 (5.7)
Employment status (%)	Employment	220 (73.3)
	Unemployment	80 (26.7)
Health status (%)	Poor	58 (19.3)
	Average	172 (57.3)
	Good	70 (23.3)
Present disease (%)	No	227 (75.7)
	Yes	73 (24.3)
Family medical history (%)	No	83 (27.7)
	Yes	217 (72.3)
Attitude toward online health video (%)	Poor	30 (10.0)
	Average	105 (35.0)
	Good	165 (55.0)
Hypertension (%)	No	262 (87.3)
	Yes	38 (12.7)
Diabetes (%)	No	292 (97.3)
	Yes	8 (2.7)
Dyslipidemia (%)	No	284 (94.7)
	Yes	16 (5.3)
Obesity (%)	No	267 (89.0)
	Yes	33 (11.0)
Intensity of social media use (median [IQR])		32.00 [28.00, 35.00]
Perceived credibility of older doctors		14.00 [12.00, 15.00]
Perceived credibility of middle-aged doctors		12.00 [11.00, 13.00]
Perceived credibility of younger doctors		11.00 [9.00, 12.00]

The median difference in perceived trustworthiness between the older and younger doctors was 3.00, with an IQR of [1.00, 5.00] (*p* < 0.001). The difference between middle-aged and younger doctors had a median of 2.00 with an IQR of [1.00, 3.00] (*p* < 0.001). The smallest difference was observed between the older and middle-aged doctors, with a median of 1.00 and an IQR of [0.00, 3.00] (*p* < 0.001) (Figure 1).

### 3.3 Relationship between social media use and age stereotypes in the condition of AI doctors

**Figure 2:**
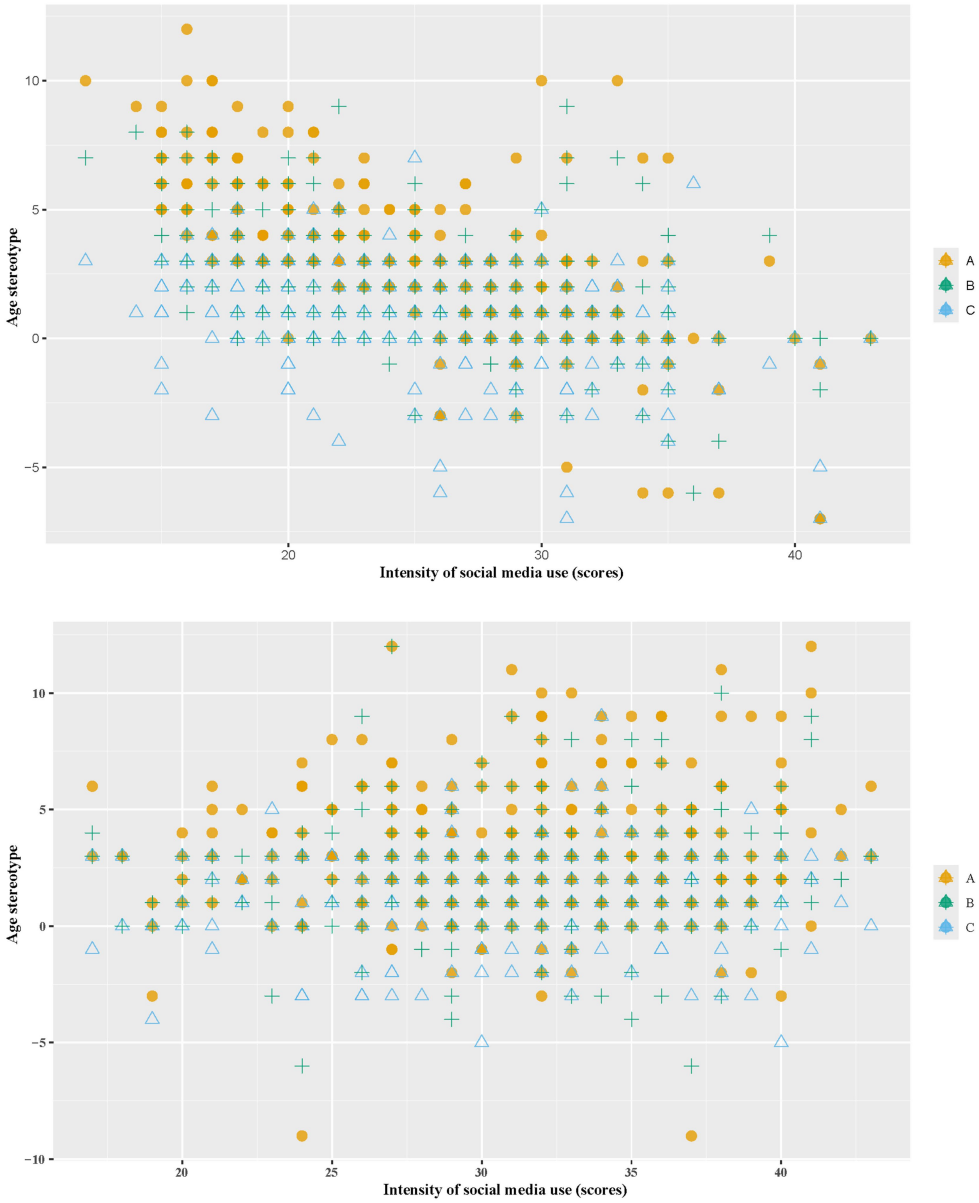
The relationship between age stereotypes and the intensity of social media usage in the condition of AI/human doctors. A, perceived credibility difference: old vs. young. B, perceived credibility difference: middle-age vs. young. C, perceived credibility difference: old vs. middle-age.

In the condition of AI doctors, in the context of AI doctors, increasing social media use was linked to a reduction in age stereotypes (Figure 2).

Table 3 showed univariable and multivariable analyses on the effect of social media use intensity on age stereotypes. The univariable analysis revealed a significant negative association between social media use and age stereotypes, particularly between older and younger doctors (*β* = −0.34, *p* < 0.001). This negative trend continued in the multivariable analysis (β = −0.32, *p* < 0.001). Similarly, the age stereotypes between middle-aged and younger doctors showed a negative association with social media use intensity (β = −0.20 in univariable, β = −0.19 in multivariable, both *p* < 0.001). The association between older and middle-aged doctors was weaker but still significant, with a multivariable β of −0.13 (*p* < 0.001). When social media use intensity was dichotomized based on the median, the association remained significant across all models (Supplementary Table S11).

**Table 3:** Relationship between social media use and age stereotypes in the condition of AI doctors.

Age stereotypes*	β (univariable)	β (multivariable)
AI		
Old vs. young	−0.34 (*P* < 0.001)	−0.32 (*P* < 0.001)^a^
Middle-age vs. young	−0.20 (*P* < 0.001)	−0.19 (*P* < 0.001)^b^
Old vs. middle-age	−0.14 (*p* < 0.001)	−0.13 (*p* < 0.001)^c^
Human		
Old vs. young	0.05 (*p* = 0.10)	0.04 (*P* = 0.17)^d^
Middle-age vs. young	0.13 (*P* = 0.17)	0.02 (*p* = 0.37)^e^
Old vs. middle-age	0.02 (*P* = 0.45)	0.02 (*p* = 0.28)^f^

*The relationship between social media use and age stereotypes was examined using generalized linear models, with social media use serving as the independent variable and age stereotypes (difference in perceived credibility among doctors of different ages) as the dependent variable.

^a^Adjusted for gender, age, monthly income, health status, and attitude toward online health video.

^b^Adjusted for age, monthly income, employment status, present disease, health status, and attitude toward online health videos.

^c^Adjusted for gender, age, health status, and attitude toward online health video.

^d^Adjusted for gender, age, monthly income, health status, and attitude toward online health video.

^e^Adjusted for gender, age, monthly income, health status, and attitude toward online health videos.

^f^Adjusted for age, educational level, health status, and attitude toward online health videos.

### 3.4 Relationship between social media use and age stereotypes in the condition of human doctors

In the condition of human doctors, as the intensity of social media use increases, age stereotypes remain largely unchanged (Figure 2).

The univariable analysis in Table 3 showed a slight, but not statistically significant, positive association between age stereotypes and social media use intensity among human doctors. Additionally, when social media use intensity was dichotomized based on the median, this association remains insignificant across all models (Supplementary Table S12).

## 4 Discussion

### 4.1 For both AI and human doctors, there is a preference for online health videos narrated by older doctors

The results of this study revealed a significant positive association between perceived credibility and the age of the doctor—both AI and human—across the three age groups (younger, middle-aged, and older) (*β* > 0, *p* < 0.001). This suggests that age stereotypes persist in online health videos regardless of whether the narrator was AI or human, with older doctors consistently perceived as more credible, thereby addressing RQ1. This finding is consistent with the conclusions of previous studies on age stereotypes among doctors (27). This suggests that AI doctors are still subject to the influence of traditional age stereotypes in building public trust.

This can be interpreted in two ways. First, individuals tend to categorize unfamiliar faces symbolically when they first encounter them (28), leading to the halo and horns effects of stereotypes (29). As a result, people are more likely to trust older doctors upon first encounter, driven by age-related stereotypes. Second, people subconsciously project their realworld experiences and perceptions onto virtual settings (30). Since age-related stereotypes about doctors are deeply embedded in people’s subconscious and shape their perceptions (31), similar biases arise when interacting with AI doctors. This insight sheds light on the deeply ingrained nature of age stereotypes and their ongoing influence in the digital healthcare setting. Additionally, it underscores the importance of addressing and mitigating potential biases when promoting AI-based medical services.

### 4.2 For AI doctors, individuals with higher intensity of social media use have weaker age stereotypes

In phase I, we found a significant negative correlation between social media use intensity and age stereotype in the AI doctor condition. Specifically, individuals with higher levels of social media use were more likely to trust younger AI doctors in online health videos. This partially answers RQ2, indicating a potential mitigating role of social media use. In previous studies, the effect of social media use on influencing stereotypes is controversial. Some researchers argue that social media use can effectively reduce stereotypes, whereas others contend that it can reinforce and even amplify stereotypes. Our results demonstrate that social media interactions can help reduce age stereotypes under certain conditions.

Age stereotypes arise from modern social interactions (32), can change stress-related emotions and hormone levels, ultimately diminishing social cognitive functions (27). Social segregation, where different age groups tend to live separately, further perpetuates age stereotypes (33). Therefore, encouraging interactions between groups is essential to reduce age stereotypes (34). Social media plays a crucial role in this regard, allowing marginalized voices to be heard and individuals to share their work, influencing public attitudes, and achieving a de-stigmatization effect (14). For example, initiatives like #ShareTheMicNowMed and blog posts by clinical doctors sharing their experiences of age stereotypes raise awareness about the challenges faced by young doctors in the medical system (35). Moreover, social media facilitates intergroup contact, fostering empathy and perspective-taking, which helps reduce prejudice (16). Video-based narratives, particularly, have been shown to diminish stereotypes and prejudice more effectively than text-based methods (16). Public service videos, like online health videos, evoke emotional responses that promote pro-social behavior (16).

This suggests that exposure to online health videos on social media can amplify this effect, weakening age stereotypes and fostering empathy toward young doctors. Therefore, advocating for young doctors through social media, especially via online health videos, can serve as an effective strategy to combat age bias and improve doctor-patient communication.

### 4.3 The mitigating role of social media use on age stereotypes was observed only in AI doctors

Comparative results from phase I and phase II further revealed that the intensity of social media use significantly reduced age stereotypes only in the AI doctor condition, while this effect was not significant in the human doctor condition. This provides a more complete answer to RQ2, highlighting that social media use may mitigate age stereotypes selectively—only when the doctor is AI-based. The AI appearance enhances the knowledge-sharing function of online health videos, thereby reducing the emphasis on doctors’ “practical experience.” As a result, the impact of social media use on reducing age stereotypes is more evident in AI doctor. This can be explained in two ways. Firstly, the trust in older doctors stems from the belief that they possess more practical experience and knowledge. For individuals who understand the logic of AI operations, these concepts do not apply to AI, and thus, they cannot assess an AI doctor’s credibility by appearance. Secondly, according to the mind perception theory (36), individuals perceive AI as capable of reasoning and performing objective tasks, thus attributing higher credibility to AI in delivering objective viewpoints compared to humans. Since online health videos primarily focus on conveying objective medical knowledge (37), those narrated by AI doctors enjoy greater credibility than those by human doctors.

This highlights a significant advantage of AI doctors. While previous researchers cautiously evaluate the effectiveness of using AI doctors (38). Our study shows that the image of AI doctors can help patients avoid being influenced by age stereotypes, enabling them to evaluate doctors’ medical expertise more objectively. This allows young doctors to transcend stereotype labels through AI images, demonstrating their medical knowledge without societal constraints. This promotes fairness and inclusivity in the medical industry, improves communication efficiency between doctors and patients, and fosters a more harmonious and efficient medical environment.

### 4.4 Practical implications

These findings also hold significant practical implications for the design of AI systems in healthcare institutions, as well as for relevant policies and technological developments.

Our study found that individuals with higher intensity of social media use have weaker age stereotype in the condition of AI doctors, For healthcare institutions, it suggest that AI doctor systems could be more widely integrated into medical practice to mitigate the trust issues between doctors and patients that arise from age stereotypes. Young doctors can also more effectively convey their professional knowledge through AI doctors.

However, our study also found that that age stereotypes regarding AI doctors still exist, hospitals should enhance the promotion of the actual capabilities of AI doctors to patients to reduce trust issues related to age. The design of hospital artificial intelligence systems should allow for operational flexibility to address potential stereotype. For example, patients could be permitted to customize the avatars of AI doctors according to their preferences, thereby preventing them from making judgments based on the preset age appearances of AI doctors. Meanwhile, considering the moderating effect of social media, AI doctors can be differentially deployed for different patient groups. For instance, older avatars of AI doctors could be provided more frequently to patients who rarely use social media, while younger avatars could be used more often for patients who frequently use social media.

Given that age stereotypes regarding AI doctors still exist, For AI developers, it is necessary to pay attention to the potential age stereotypes caused by AI doctors in visual design. First, background visual cues can be employed, such as matching young AI doctors with virtual white coats featuring the badge of “Chief Physician” which can enhance the perceived authority of young AI doctors. Second, moderately neutral appearance designs should be adopted to avoid overemphasizing age-related features and to strengthen professional identity cues (e.g., qualifications, case numbers, and satisfaction rates), thereby shifting patients’ attention to professional knowledge.

At the policy level, healthcare regulatory bodies should establish guidelines to ensure that the appearance, functionality, and communication of AI doctors are in line with the principles of scientific communication. They should also further promote the digitalization of healthcare and the integration of social media with digital health services, especially for digital disadvantaged groups such as old patients in rural areas. Additionally, developers should be required to disclose algorithmic fairness standards to prevent the reinforcement of age stereotypes through visual design.

### 4.5 Limitations

There are potential limitations to extending our findings. Firstly, participants volunteered to complete the survey, potentially introducing selection bias. To reduce non-response bias in future studies, efforts could be made to recruit participants through more comprehensive outreach methods, such as following up with non-respondents, offering incentives, using multiple survey modes, and addressing potential barriers to participation. Secondly, all participants were from East Asia, necessitating caution regarding the generalizability of our results. In order to enhance the generalizability, follow-up research could be conducted with more diverse participant samples that include individuals from various regions and cultural backgrounds. Third, while we adopted various measures to enhance the reliability and validity of the results, however, it’s important to recognize that completely eliminating self-reporting bias remains a challenge. To further minimize this bias, future research might consider incorporating additional data collection methods, such as objective measurements or third-party observations, in conjunction with self-reports. Longitudinal studies should be conducted in the future to assess the long-term integration effects of AI in healthcare settings, including its impact on trust between doctors and patients, satisfaction levels, and health outcomes over time.

## Data Availability

The raw data supporting the conclusions of this article will be made available by the authors, without undue reservation.
