# Supplementary material for: Credibility of AI generated and human video doctors and the relationship to social media use
Source: Front Public Health. 2025 Jul 9;13:1559378. doi: 10.3389/fpubh.2025.1559378 (PMC12283982; doi:10.3389/fpubh.2025.1559378)
Supplement: Supplementary file 1 [file Table_1.docx]

**Supplementary material**

[**Questionnaire 1** 2](#_Toc179063453)

[**Questionnaire 2** 5](#_Toc179063454)

[Table S1 Intensity of social media use scale 8](#_Toc179063455)

[Table S2 Perceived credibility scale 9](#_Toc179063456)

[Table S3 Univariable linear regression results in in the condition of AI doctors (Old vs young) 10](#_Toc179063457)

[Table S4 Univariable linear regression results in in the condition of AI doctors (Middle-age vs young) 11](#_Toc179063458)

[Table S5 Univariable linear regression results in in the condition of AI doctors (Old vs Middle-age) 12](#_Toc179063459)

[Table S6 Univariable linear regression results in in the condition of Human doctors (Old vs young) 13](#_Toc179063460)

[Table S7 Univariable linear regression results in in the condition of human doctors (Middle-age vs young) 14](#_Toc179063461)

[Table S8 Univariable linear regression results in in the condition of human doctors (Old vs Middle-age) 15](#_Toc179063462)

[Table S9 Reliability and validity in the condition of AI 16](#_Toc179063463)

[Table S10 Reliability and validity in the condition of human 17](#_Toc179063464)

[Table S11 Sensitivity analysis in the condition of AI doctors 18](#_Toc179063465)

[Table S12 Sensitivity analysis in the condition of human doctors 19](#_Toc179063466)

**Questionnaire 1**

Patient ID  Informed Consent □ Hospital of Visit：

Contact Information

**Section One: Basic Information**

Gender male □ female □ Age  years Height  cm

Weight .kg

**Section Two: Education Level**

Elementary school □ Junior high school□ Senior high school □

Associate degree □ Bachelor’s degree □ Master’s degree or above □

**Section Three: Marital status**

Widowed/Divorced/Separated/Never married □ Married/Living with partner □

**Section Four: Monthly Income**

<3000 yuan □ 3000-8000 yuan □ 8000-20000 yuan □ >20000 yuan □

**Section Five: Employment Status**

Employment □ Unemployment □

**Section Six: Health Status**

Poor □ Average □ Good □

**Section Seven: Disease**

Yes □ No □

Hypertension □ Diabetes □ Dyslipidemia □ Obesity □ Others □

**Section Eight: Family History**

Yes □ No □

Hypertension □ Diabetes □ Dyslipidemia □ Obesity □ Others □

**Section Nine: Acceptance of Online Health video**

Strongly Disagree □ Disagree □ Neutral □ Agree □ Strongly Agree □

Note: “Strongly Disagree”, “Disagree” and “Neutral” were under the category of “Poor”. “Agree” was under the category of “Average”, “Strongly Agree” was under the category of “Good”

**Section Ten: Evaluation of the speaker's perceived credibility**


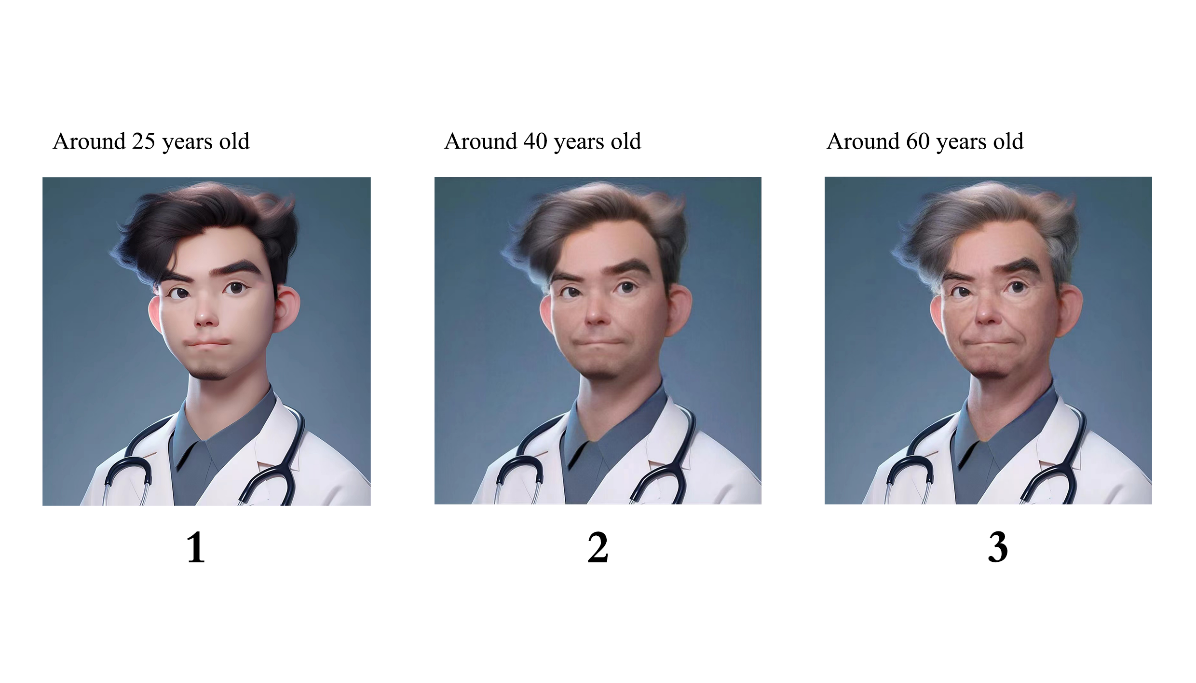


Note: The theme of the videos revolves around a range of everyday health knowledge, including but not limited to: “Whether to add or remove clothing during fever”, “How to improve sleep quality”, “Effective measures for the prevention and control of myopia”, “Proper techniques for measuring body temperature and blood pressure”, and “Scientific approaches to exercise”.

Do you think these doctors are AI doctors or real human doctors?

AI □ Human □

**Perceived credibility scale**

| Individual Items | Score | The score of Doctor No. 1 | The score of Doctor No. 1 | The score of Doctor No. 1 |
| --- | --- | --- | --- | --- |
| I think the doctor is credible | 5 = strongly approve, 4 = approve, 3=undecided,2 = disapprove, and 1 = strongly disapprove. |  |  |  |
| I think the doctor is true | 5 = strongly approve, 4 = approve, 3=undecided,2 = disapprove, and 1 = strongly disapprove. |  |  |  |
| I think the doctor is reliable | 5 = strongly approve, 4 = approve, 3=undecided,2 = disapprove, and 1 = strongly disapprove. |  |  |  |

**Section Eleven:** **social media use**

| Individual Items | Score |
| --- | --- |
| About how many total friends do you have on the most frequently used social media platform for watching short videos? | 0 = 10 or less, 1 = 11–50, 2 = 51–100, 3 = 101–150, 4 = 151–200, 5 = 201–250, 6 = 251–300, 7 = 301–400, 8 = more than 400 |
| In the past week, on average, approximately how many minutes per day have you spent on the most frequently used social media platform for watching short videos? | 0 = less than 10, 1 = 10–30, 2 = 31–60, 3 = 1–2 hours, 4 = 2–3 hours, 5 = more than 3 hours |
| The most frequently used social media platform is part of my everyday activity | 5 = strongly approve, 4 = approve, 3=undecided,2 = disapprove, and 1 = strongly disapprove. |
| I am proud to tell people I’m on the most frequently used social media platform | 5 = strongly approve, 4 = approve, 3=undecided,2 = disapprove, and 1 = strongly disapprove. |
| The most frequently used social media platform has become part of my daily routine | 5 = strongly approve, 4 = approve, 3=undecided,2 = disapprove, and 1 = strongly disapprove. |
| I feel out of touch when I haven’t logged onto the most frequently used social media platform for a while | 5 = strongly approve, 4 = approve, 3=undecided,2 = disapprove, and 1 = strongly disapprove. |
| I feel I am part of the most frequently used social media platform community | 5 = strongly approve, 4 = approve, 3=undecided,2 = disapprove, and 1 = strongly disapprove. |
| I would be sorry if the most frequently used social media platform shut down | 5 = strongly approve, 4 = approve, 3=undecided,2 = disapprove, and 1 = strongly disapprove. |

**Questionnaire 2**

Patient ID  Informed Consent □ Hospital of Visit：

Contact Information

**Section One: Basic Information**

Gender male □ female □ Age  years Height  cm

Weight .kg

**Section Two: Education Level**

Elementary school □ Junior high school□ Senior high school □

Associate degree □ Bachelor’s degree □ Master’s degree or above □

**Section Three: Marital status**

Widowed/Divorced/Separated/Never married □ Married/Living with partner □

**Section Four: Monthly Income**

<3000 yuan □ 3000-8000 yuan □ 8000-20000 yuan □ >20000 yuan □

**Section Five: Employment Status**

Employment □ Unemployment □

**Section Six: Health Status**

Poor □ Average □ Good □

**Section Seven: Disease**

Yes □ No □

Hypertension □ Diabetes □ Dyslipidemia □ Obesity □ Others □

**Section Eight: Family History**

Yes □ No □

Hypertension □ Diabetes □ Dyslipidemia □ Obesity □ Others □

**Section Nine: Acceptance of Online Health video**

Strongly Disagree □ Disagree □ Neutral □ Agree □ Strongly Agree □

Note: “Strongly Disagree”, “Disagree” and “Neutral” were under the category of “Poor”. “Agree” was under the category of “Average”, “Strongly Agree” was under the category of “Good”

**Section Ten: Evaluation of the speaker's perceived credibility**


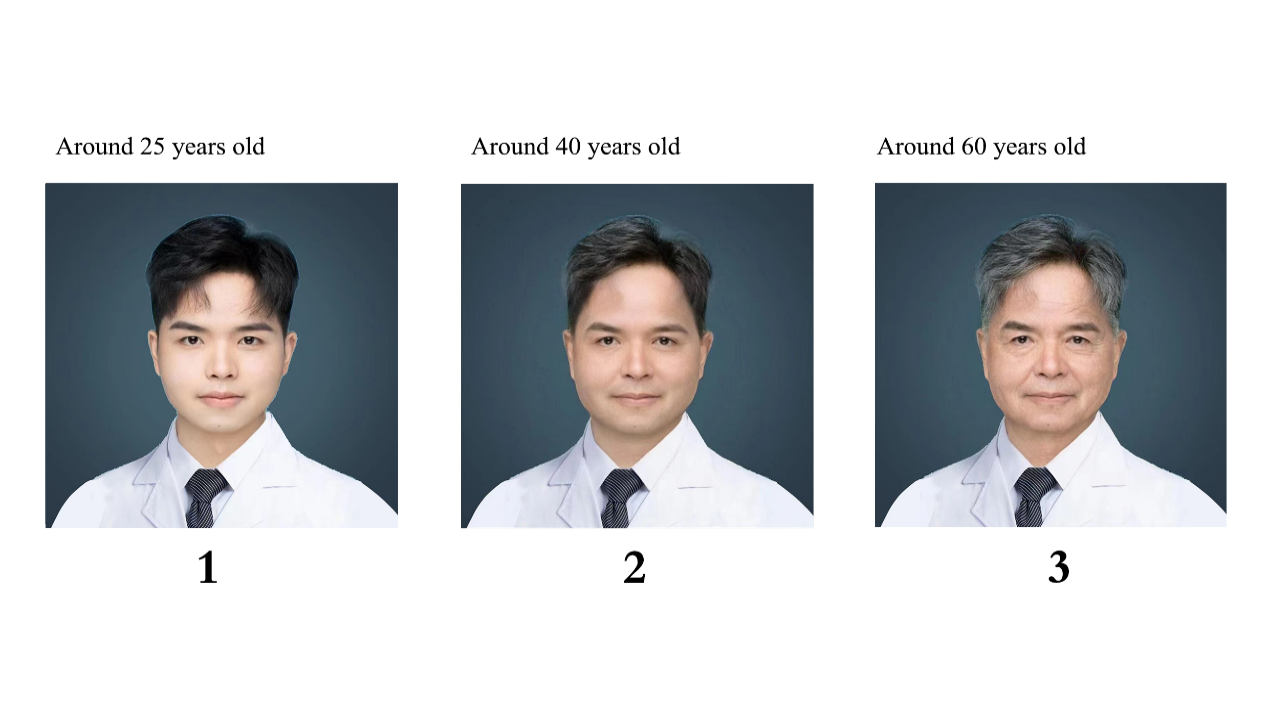


Note: The theme of the videos revolves around a range of everyday health knowledge, including but not limited to: “Whether to add or remove clothing during fever”, “How to improve sleep quality”, “Effective measures for the prevention and control of myopia”, “Proper techniques for measuring body temperature and blood pressure”, and “Scientific approaches to exercise”.

Do you think these doctors are AI doctors or real human doctors?

AI □ Human □

**Perceived credibility scale**

| Individual Items | Score | The score of Doctor No. 1 | The score of Doctor No. 1 | The score of Doctor No. 1 |
| --- | --- | --- | --- | --- |
| I think the doctor is credible | 5 = strongly approve, 4 = approve, 3=undecided,2 = disapprove, and 1 = strongly disapprove. |  |  |  |
| I think the doctor is true | 5 = strongly approve, 4 = approve, 3=undecided,2 = disapprove, and 1 = strongly disapprove. |  |  |  |
| I think the doctor is reliable | 5 = strongly approve, 4 = approve, 3=undecided,2 = disapprove, and 1 = strongly disapprove. |  |  |  |

**Section Eleven: social media use**

| Individual Items | Score |
| --- | --- |
| About how many total friends do you have on the most frequently used social media platform for watching short videos? | 0 = 10 or less, 1 = 11–50, 2 = 51–100, 3 = 101–150, 4 = 151–200, 5 = 201–250, 6 = 251–300, 7 = 301–400, 8 = more than 400 |
| In the past week, on average, approximately how many minutes per day have you spent on the most frequently used social media platform for watching short videos? | 0 = less than 10, 1 = 10–30, 2 = 31–60, 3 = 1–2 hours, 4 = 2–3 hours, 5 = more than 3 hours |
| The most frequently used social media platform is part of my everyday activity | 5 = strongly approve, 4 = approve, 3=undecided,2 = disapprove, and 1 = strongly disapprove. |
| I am proud to tell people I’m on the most frequently used social media platform | 5 = strongly approve, 4 = approve, 3=undecided,2 = disapprove, and 1 = strongly disapprove. |
| The most frequently used social media platform has become part of my daily routine | 5 = strongly approve, 4 = approve, 3=undecided,2 = disapprove, and 1 = strongly disapprove. |
| I feel out of touch when I haven’t logged onto the most frequently used social media platform for a while | 5 = strongly approve, 4 = approve, 3=undecided,2 = disapprove, and 1 = strongly disapprove. |
| I feel I am part of the most frequently used social media platform community | 5 = strongly approve, 4 = approve, 3=undecided,2 = disapprove, and 1 = strongly disapprove. |
| I would be sorry if the most frequently used social media platform shut down | 5 = strongly approve, 4 = approve, 3=undecided,2 = disapprove, and 1 = strongly disapprove. |

# Table S1 Intensity of social media use scale

| Individual Items | Score |
| --- | --- |
| About how many total friends do you have on the most frequently used social media platform for watching short videos? | 0 = 10 or less, 1 = 11–50, 2 = 51–100, 3 = 101–150, 4 = 151–200, 5 = 201–250, 6 = 251–300, 7 = 301–400, 8 = more than 400 |
| In the past week, on average, approximately how many minutes per day have you spent on the most frequently used social media platform for watching short videos? | 0 = less than 10, 1 = 10–30, 2 = 31–60, 3 = 1–2 hours, 4 = 2–3 hours, 5 = more than 3 hours |
| The most frequently used social media platform is part of my everyday activity | 5 = strongly approve, 4 = approve, 3=undecided,2 = disapprove, and 1 = strongly disapprove· |
| I am proud to tell people I am on the most frequently used social media platform | 5 = strongly approve, 4 = approve, 3=undecided,2 = disapprove, and 1 = strongly disapprove· |
| The most frequently used social media platform has become part of my daily routine | 5 = strongly approve, 4 = approve, 3=undecided,2 = disapprove, and 1 = strongly disapprove· |
| I feel out of touch when I haven’t logged onto the most frequently used social media platform for a while | 5 = strongly approve, 4 = approve, 3=undecided,2 = disapprove, and 1 = strongly disapprove· |
| I feel I am part of the most frequently used social media platform community | 5 = strongly approve, 4 = approve, 3=undecided,2 = disapprove, and 1 = strongly disapprove· |
| I would be sorry if the most frequently used social media platform shut down | 5 = strongly approve, 4 = approve, 3=undecided,2 = disapprove, and 1 = strongly disapprove· |

# Table S2 Perceived credibility scale

| Individual Items | Score | The score of Doctor No· 1 | The score of Doctor No· 2 | The score of Doctor No· 3 |
| --- | --- | --- | --- | --- |
| I think the doctor is credible | 5 = strongly approve, 4 = approve, 3=undecided,2 = disapprove, and 1 = strongly disapprove· |  |  |  |
| I think the doctor is true | 5 = strongly approve, 4 = approve, 3=undecided,2 = disapprove, and 1 = strongly disapprove· |  |  |  |
| I think the doctor is reliable | 5 = strongly approve, 4 = approve, 3=undecided,2 = disapprove, and 1 = strongly disapprove· |  |  |  |

# Table S3 Univariable linear regression results in in the condition of AI doctors (Old vs young)

| Variables | β (univariable) |
| --- | --- |
| Gender  Male  Female* | Reference  -**0·84 (P = 0·02)** |
| Age  16-24  25-35  35-43  44-69 | Reference  -0·28 (P = 0·55)  -0·10 (P = 0·85)  0·36 (P = 0·46) |
| BMI | 0·02 (P = 0·69) |
| Education  Elementary school  Junior high school  Senior high school  Associate degree  Bachelor’s degree  Master’s degree or above | Reference  1·16 (P = 0·11)  0·84 (P = 0·24)  1·27 (P = 0·09)  0·79 (P = 0·25)  0·17 (P = 0·82) |
| Marital status  Widowed/Divorced/Separated/Never married  Married/Living with partner | 0·11 (P = 0·76) |
| Monthly income  <3000 yuan  3000-8000 yuan  8000-20000 yuan  >20000 yuan | Reference  **1·33 (P = 0·004)**  **0·93 (P = 0·04)**  0·79 (P = 0·29) |
| Employment status  Employment  Unemployment | Reference  -0·69 (P = 0·08) |
| Health status  Poor  Average  Good | Reference  -0·36 (P = 0·40)  -0·19 (P = 0·69) |
| Present disease  No  Yes | Reference  0·74 (P = 0·054) |
| Family medical history  No  Yes | Reference  -0·48 (P = 0·21) |
| Attitude towards online health video  Poor  Average  Good | Reference  **1·06 (P = 0·04)**  **1·83 (P < 0·001)** |

# Table S4 Univariable linear regression results in in the condition of AI doctors (Middle-age vs young)

| Variables | β (univariable) |
| --- | --- |
| Gender  Male  Female* | Reference  -0·20 (P = 0·49) |
| Age  16-24  25-35  35-43  44-69 | Reference  -0·01 (P = 0·97)  0·61 (P = 0·12)  -0·11 (P = 0·77) |
| BMI | 0·02 (P = 0·66) |
| Education  Elementary school  Junior high school  Senior high school  Associate degree  Bachelor’s degree  Master’s degree or above | Reference  0·60 (P = 0·29)  0·55 (P = 0·33)  0·73 (P = 0·21)  0·47 (P = 0·39)  0·09 (P = 0·88) |
| Marital status  Widowed/Divorced/Separated/Never married  Married/Living with partner | 0·22 (P = 0·43) |
| Monthly income  <3000 yuan  3000-8000 yuan  8000-20000 yuan  >20000 yuan | Reference  **1·09 (P = 0·002)**  **0·78 (P = 0·03)**  0·34 (P = 0·57) |
| Employment status  Employment  Unemployment | Reference  **0·60 (P = 0·04**) |
| Health status  Poor  Average  Good | Reference  0·24 (P = 0·47)  0·29 (P = 0·46) |
| Present disease  No  Yes | Reference  0**·92 (P = 0·002)** |
| Family medical history  No  Yes | Reference  -0·11 (P = 0·71) |
| Attitude towards online health video  Poor  Average  Good | Reference  0·63 (P = 0·12)  **0·97 (P = 0·01)** |

# Table S5 Univariable linear regression results in in the condition of AI doctors (Old vs Middle-age)

| Variables | β (univariable) |
| --- | --- |
| Gender  Male  Female* | Reference  **-0·64 (P = 0·01)** |
| Age  16-24  25-35  35-43  44-69 | Reference  -0·27 (P = 0·41)  0·51 (P = 0·14)  0·47 (P = 0·16) |
| BMI | 0·00 (P = 0·93) |
| Education  Elementary school  Junior high school  Senior high school  Associate degree  Bachelor’s degree  Master’s degree or above | Reference  0·56 (P = 0·27)  0·29 (P = 0·57)  0·54 (P = 0·30)  0·32 (P = 0·51)  0·08 (P = 0·87) |
| Marital status  Widowed/Divorced/Separated/Never married  Married/Living with partner | -0·11 (P = 0·65) |
| Monthly income  <3000 yuan  3000-8000 yuan  8000-20000 yuan  >20000 yuan | Reference  0·24 (P = 0·45)  0·15 (P = 0·65)  0·45 (P = 0·39) |
| Employment status  Employment  Unemployment | Reference  -0·09 (P = 0·75) |
| Health status  Poor  Average  Good | Reference  **0·60 (P = 0·04)**  -0·48 (P = 0·16) |
| Present disease  No  Yes | Reference  -0·18 (P = 0·50) |
| Family medical history  No  Yes | Reference  -0·36 (P = 0·17) |
| Attitude towards online health video  Poor  Average  Good | Reference  0·43 (P = 0·23)  **0·85 (P = 0·02)** |

# Table S6 Univariable linear regression results in in the condition of Human doctors (Old vs young)

| Variables | β (univariable) |
| --- | --- |
| Gender  Male  Female* | Reference  **-1·02 (P = 0·04)** |
| Age  18-24  25-33  34-43  44-65 | Reference  -0·28 (P = 0·54)  0·17 (P = 0·72)  0·48 (P = 0·31) |
| BMI | -0·02 (P = 0·62) |
| Education  Elementary school  Junior high school  Senior high school  Associate degree  Bachelor’s degree  Master’s degree or above | Reference  -0·31 (P = 0·74)  -1·02 (P = 0·29)  -1·60 (P = 0·11)  -1·11 (P = 0·25)  -1·65 (P = 0·13) |
| Marital status  Widowed/Divorced/Separated/Never married  Married/Living with partner | 0·39 (P = 0·25) |
| Monthly income  <3000 yuan  3000-8000 yuan  8000-20000 yuan  >20000 yuan | Reference  **1·13 (P = 0·01)**  0·30 (P = 0·50)  1·20 (P = 0·12) |
| Employment status  Employment  Unemployment | Reference  -0·25 (P = 0·51) |
| Health status  Poor  Average  Good | Reference  0·27 (P = 0·53)  0·05 (P = 0·92) |
| Present disease  No  Yes | Reference  0·29 (P = 0·45) |
| Family medical history  No  Yes | Reference  -0·15 (P = 0·69) |
| Attitude towards online health video  Poor  Average  Good | Reference  -0·29 (P = 0·63)  -0·45 (P = 0·43) |

# Table S7 Univariable linear regression results in in the condition of human doctors (Middle-age vs young)

| Variables | β (univariable) |
| --- | --- |
| Gender  Male  Female* | Reference  -**0·73 (P = 0·01)** |
| Age  18-24  25-33  34-43  44-65 | Reference  0·44 (P = 0·24)  0·26 (P = 0·49)  0·34 (P = 0·38) |
| BMI | 0·00 (P = 0·93) |
| Education  Elementary school  Junior high school  Senior high school  Associate degree  Bachelor’s degree  Master’s degree or above | Reference  0·70 (P = ·38)  0·54 (P = ·49)  0·42 (P = ·61)  0·55 (P = ·49)  -0·15 (P = ·87) |
| Marital status  Widowed/Divorced/Separated/Never married  Married/Living with partner | 0·31 (P = ·26) |
| Monthly income  <3000 yuan  3000-8000 yuan  8000-20000 yuan  >20000 yuan | Reference  **1·15 (P = 0·002)**  0·52 (P = 0·16)  0·80 (P = 0·21) |
| Employment status  Employment  Unemployment | Reference  -0·54 (P = 0·08) |
| Health status  Poor  Average  Good | Reference  -0·05 (P = 0·89)  0·23 (P = 0·58) |
| Present disease  No  Yes | Reference  -0·15 (P = 0·64) |
| Family medical history  No  Yes | Reference  0·20 (P = 0·51) |
| Attitude towards online health video  Poor  Average  Good | Reference  -0·10 (P = 0·84)  -0·19 (P = 0·69) |

# Table S8 Univariable linear regression results in in the condition of human doctors (Old vs Middle-age)

| Variables | β (univariable) |
| --- | --- |
| Gender  Male  Female* | Reference  -0·29 (P = 0·22) |
| Age  18-24  25-33  34-43  44-65 | Reference  **-0·72 (P = 0·02)**  -0·10 (P = 0·76)  0·14 (P = 0·66) |
| BMI | -0·03 (P = 0·39) |
| Education  Elementary school  Junior high school  Senior high school  Associate degree  Bachelor’s degree  Master’s degree or above | Reference  -1·01 (P = 0·11)  **1·56 (P = 0·01)**  **-2·03 (P = 0·002)**  **-1·66 (P = 0·009)**  **-1·51 (P = 0·03)** |
| Marital status  Widowed/Divorced/Separated/Never married  Married/Living with partner | 0·08 (P = 0·73) |
| Monthly income  <3000 yuan  3000-8000 yuan  8000-20000 yuan  >20000 yuan | Reference  -0·02 (P = 0·96)  -0·22 (P = 0·48)  0·40 (P = 0·44) |
| Employment status  Employment  Unemployment | Reference  0·29 (P = 0·25) |
| Health status  Poor  Average  Good | Reference  0·33 (P = 0·26)  0·18 (P = 0·59) |
| Present disease  No  Yes | Reference  -0·14 (P = 0·58) |
| Family medical history  No  Yes | Reference  -0·06 (P = 0·81) |
| Attitude towards online health video  Poor  Average  Good | Reference  -0·19 (P = 0·63)  -0·25 (P = 0·50) |

# Table S9 Reliability and validity in the condition of AI

| Scales | Cronbach’s α coefficients | KMO value |
| --- | --- | --- |
| Perceived credibility | 0·81 (Young)  0·72 (Middle age)  0·84 (Old) | 0·69 (Young)*  0·67 (Middle-age)*  0·71 (Old)* |
| Intensity of social media use | 0·62 | 0·82* |

**P*<0·05

# Table S10 Reliability and validity in the condition of human

| Scales | Cronbach’s α coefficients | KMO value |
| --- | --- | --- |
| Perceived credibility | 0·82 (Young)  0·75 (Middle age)  0·74 (Old) | 0·71 (Young)*  0·68 (Middle-age)*  0·67 (Old)* |
| Intensity of social media use | 0·61 | 0·82* |

**P*<0·05

# Table S11 Sensitivity analysis in the condition of AI doctors

| Age stereotypes | β (univariable) | β (multivariable) |
| --- | --- | --- |
| Old *vs* young | -3·59 (*P* < 0·001) | -3·38 (*P* < 0·001)^a^ |
| Middle-age *vs* young | -2·00 (*P* < 0·001) | -1·82 (*P* < 0·001)^b^ |
| Old *vs* Middle-age | -1·59 (*P* < 0·001) | -1·50 (*P* < 0·001)^c^ |

^a^Adjusted for gender, age, monthly income, health status, and attitude towards online health video.

^b^ Adjusted for age, monthly income, employment status, present disease, health status, and attitude towards online health videos.

^c^ Adjusted for gender, age, health status, and attitude towards online health video.

All results are based on the lower intensity of social media use group as the reference group

# Table S12 Sensitivity analysis in the condition of human doctors

| Age stereotypes | β (univariable) | β (multivariable) |
| --- | --- | --- |
| Old *vs* young | 0·44 (*P* = 0·19) | 0·37 (*P* = 0·28)^a^ |
| Middle-age *vs* young | 0·18(*P* = 0·49) | 0·09 (*P* = 0·76)^b^ |
| Old *vs* Middle-age | 0·25 (*P* = 0·26) | 0·26 (*P* = 0·24)^c^ |

^a^Adjusted for gender, age, monthly income, health status, and attitude towards online health video.

^b^ Adjusted for gender, age, monthly income, health status, and attitude towards online health videos.

^c^ Adjusted for age, educational level, health status, and attitude towards online health videos.

All results are based on the lower intensity of social media use group as the reference group
